# Dynamical Multimodality in Systems Driven by Ornstein–Uhlenbeck Noise

**DOI:** 10.3390/e27030263

**Published:** 2025-03-02

**Authors:** Michał Mandrysz, Bartłomiej Dybiec

**Affiliations:** 1 Faculty of Physics, Astronomy and Applied Computer Science, Jagiellonian University, ul. St. Łojasiewicza 11, 30-348 Kraków, Poland; 2 Institute of Theoretical Physics, and Mark Kac Center for Complex Systems Research, Jagiellonian University, ul. St. Łojasiewicza 11, 30-348 Kraków, Poland

**Keywords:** Ornstein–Uhlenbeck process, dichotomous noise, stationary density, stochastic dynamics

## Abstract

The properties of dynamical systems driven by noise are determined by the combined action of deterministic forces and random fluctuations. The action of non-white (correlated) noise is capable of producing stationary states with a number of modes larger than the number of (stable) fixed points of the deterministic potential. In particular, the action of Ornstein–Uhlenbeck noise can induce the bimodality of the stationary states in fixed single-well potentials. Here, we study the emergence of dynamical multimodality in systems subject to the simultaneous action of Ornstein–Uhlenbeck and Markovian dichotomous noise in 1D and 2D setups. The randomization of the potential due to the action of dichotomous noise can be used to control the number of modes in the stationary states.

## 1. Introduction

The interaction of a test particle with its surrounding environment, often complex and unknown, can be effectively modeled by noise [1], which provides a crucial component of the Langevin equation. A common first approximation assumes that the noise is both Gaussian and white. However, these simplifications are not always applicable. Numerous deviations from Gaussianity have been observed [2,3], and noise may exhibit temporal correlations, i.e., it could be non-white [1].

In this study, we analyze the dynamics of anharmonic stochastic oscillators subject to the action of Ornstein–Uhlenbeck (OU) noise [4,5]. The Ornstein–Uhlenbeck process is a Gaussian, Markovian stochastic process characterized by an exponentially decaying autocorrelation function. The non-whiteness, inherent in this type of noise, plays a crucial role in the emergence of bimodal stationary probability distributions within single-well superharmonic potentials. In particular, the action of Ornstein–Uhlenbeck noise can produce bimodal stationary states in fixed quartic potentials. Here, we verify if the additional action of Markovian dichotomous noise can affect the modality of stationary states in single-well potentials in comparison to the pure OU driving.

The analytical treatment of systems driven by non-white noise presents a significant challenge compared with the analysis of systems subject to Gaussian white noise. This fact calls for the development and application of various approximate and numerical methods. For systems driven by OU noise, a variety of approximate analytical techniques have been proposed and explored [6,7,8,9,10,11,12]. The unified colored noise approximation [12] plays a particularly important role due to its applicability for both small and large correlation times and its broad range of validity with respect to the noise intensity.

Multimodal stationary states can arise in single-well, static potentials through two distinct, well-known mechanisms. One mechanism is based on the temporal correlations present in colored noise, while the other relies on the characteristics of the jump length distribution. In the case of Ornstein–Uhlenbeck and fractional Gaussian noises, multimodality emerges from the interplay between correlated, noise-driven motion [13,14,15] and the deterministic restoring force. This mechanism will be examined in detail for motions in randomized potentials studied in subsequent sections. The other mechanism allowing for the emergence of bimodal stationary states is based on Lévy flights. Lévy flights induce bimodality through a fundamentally different mechanism [16,17,18]. This mechanism exploits the heavy-tailed asymptotics of the jump length distribution, enabling anomalously long jumps. Lévy noise is frequently employed to model out-of-equilibrium systems [2,19] exhibiting heavy-tailed fluctuations [20,21,22,23,24,25,26,27,28,29]. Despite their distinct origins, these two mechanisms share a common feature. In sufficiently steep potential wells, both can lead to a depletion in probability density near the potential minimum. This depletion, whether caused by correlated (persistent) motion or by long jumps, results in the emergence of bimodal stationary distributions in overdamped systems.

In Section 2, the model under the study is presented. Within Section 2.1, we recall basic information regarding dynamics in the parabolic and quartic potentials under the action of Ornstein–Uhlenbeck noise. Finally, in Section 2.2, we study the combined action of Ornstein–Uhlenbeck and Markovian dichotomous noise types in 1D (Section 2.2.1) and 2D (Section 2.2.2) setups, demonstrating that the randomization of the potential can further change the modality of stationary states.

## 2. Model and Results

The overdamped Langevin equation

(1) $$\dot{x}\left(t\right)=-V^{\prime }\left(x,t\right)+\eta \left(t\right).$$

underlines the versatility of models. Here, we study the properties of overdamped, noise-perturbed motion in an external, time-dependent potential V(x,t). It is assumed that the noise η(t) in Equation (1) is of the Ornstein–Uhlenbeck type, i.e., the motion described by Equation (1) is driven by Markovian, non-white noise. Consequently, the Langevin equation is accompanied by another equation describing the evolution of noise. The Ornstein–Uhlenbeck process satisfies the following stochastic differential equation:

(2) $$\dot{\eta }\left(t\right)=-\rho \;\eta \left(t\right)+\rho \sqrt{2D}\;\xi \left(t\right),$$

where ξ(t) stands for the delta-correlated Gaussian white noise with unit intensity 〈ξ(t)ξ(t+τ)〉=δ(τ) and zero mean 〈ξ(t)〉=0. The Ornstein–Uhlenbeck process [4] has the exponentially decaying autocorrelation function, which, for η(0)=0, is given by

(3) $$\langle \eta \left(t\right)\eta \left(s\right)\rangle =D\rho \left[e^{-\rho |s-t|}-e^{-\rho (s+t)}\right].$$

The correlation time τ of the Ornstein–Uhlenbeck process is τ=1/ρ. In the literature, there are other conventions defining the OU process. We use the one defined by Equation (2), which is also used in [12]. The advantage of such a convention is the fact that in the ρ→∞ limit and equivalently the τ=1/ρ→0 limit, the Ornstein–Uhlenbeck noise η(t) becomes Gaussian white noise with intensity 2D.

The OU process (see Equation (2)) is closely related to the motion of a free particle ($\dot{x}=v$) subjected to linear damping and stochastic (Gaussian white noise) perturbation ($\dot{v}=-\gamma v+\xi$); see [30]. In such a case, the position can be interpreted as the integrated OU process [31]. It also appears naturally in the description of Brownian motion in the harmonic potential ($\dot{x}=-kx+\xi$). Here, the OU process is applied as a more general process that can be used to replace the Gaussian white noise [32,33,34,35] in the overdamped Langevin equation; see Equation (1).

### 2.1. Test Bench: Static Potentials

In most cases, the Langevin equation (Equation (1)) can be studied numerically only. From the Langevin equation, one can generate multiple realizations of the stochastic process governed by Equation (1), from which time-dependent densities P(x,t) can be estimated. The required realizations of the process x(t) can be approximated by the Euler–Maruyama method [36,37]. In order to generate x(t), it is necessary to generate consecutive values of the OU process as

(4) $$\eta \left(t+\Delta t\right)=\eta \left(t\right)-\rho \eta \left(t\right)\Delta t+\rho \sqrt{2D}\sqrt{\Delta t}\xi_{t},$$

where $\xi_{t}$ is the sequence of independent identically distributed random variables following Gaussian (normal) N(0,1) density. From η(t), the desired x(t) is calculated trajectorywise:

(5) $$x\left(t+\Delta t\right)=x\left(t\right)-V^{\prime }\left(x\left(t\right)\right)\Delta t+\eta \left(t\right)\Delta t.$$

Numerous trajectories allow for the calculation of different characteristics and quantifiers defined on the stochastic process, indicating the crucial role played by numerical methods allowing for the generation of sample realizations of Equation (1). These methods need to be thoroughly tested. Such extensive testing has been performed in a series of earlier works [38,39,40]. These tests included the exploration of stationary states, e.g., in the parabolic case, as well as the exploration of time-dependent densities and characteristics. The basic set of tests were performed for the parabolic potential, as it is analytically tractable [40]. The performed tests confirmed that the numerical integration of stochastic ordinary differential equations driven by OU noise (see Equation (1)) is correctly implemented and thus can be used for further studies.

The overdamped OU noise-driven motion in a static potential V(x) is described by the following Smoluchowski–Fokker–Planck equation [4,5]:

(6) $$\frac{\partial P}{\partial t}=\frac{\partial }{\partial x}\left[(V^{\prime }\left(x\right))P\right]-\frac{\partial }{\partial x}\left(\eta P\right)+\rho \frac{\partial }{\partial \eta }\left(\eta P\right)+\rho^{2}D\frac{\partial^{2}}{\partial \eta^{2}}P,$$

where P=P(x,η,t|x(0),η(0),0). For the parabolic potential $V\left(x\right)=ax^{2}/2$ (with the fixed initial conditions x(0) and η(0)), the solution of the diffusion equation is given by the 2D normal distribution in (x,η). The case of the quartic potential

(7) $$V\left(x\right)=\frac{b}{4}x^{4}+\frac{a}{2}x^{2}$$

is not fully traceable analytically, but it can be studied numerically. It is also accessible to some approximate methods [9,12,13,41]. The unified colored noise approximation [12] gives the formula for stationary density as

(8) $$P\left(x\right)\propto e^{-V(x)/D}\left|1+\tau V^{\prime \prime }\left(x\right)\right|e^{-\tau {V^{\prime }}^{2}\left(x\right)/\left(2D\right)},$$

which is valid for small and large correlation times [12] and a broad spectrum of noise intensities. By using the unified colored noise approximation (UCNA) [12], it is possible to demonstrate that Ornstein–Uhlenbeck noise can produce bimodal stationary states [13,40] in single-well quartic potentials (a>0,b>0) when

(9) $$a{\left(1+a\tau \right)}^{2}<6Db\tau .$$

Otherwise, stationary states are unimodal [13,40]. Equation (9) implies that for the (pure) quartic potential, i.e., quartic potential (7) with a=0, according to the UCNA, the stationary state is always bimodal.

**Figure 1:**
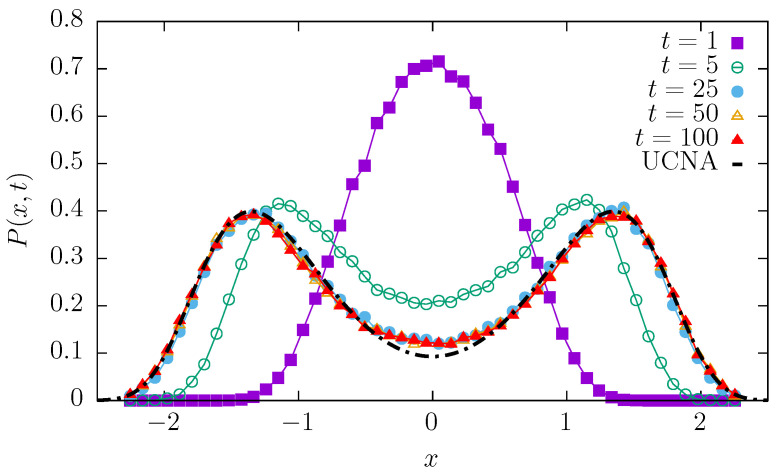
Time-dependent densities P(x,t) at various times for the single-well quartic potential (a=1 and b=1) with ρ=0.05 and D= 40,000. The dot-dashed line depicts the UCN approximation given by Equation (8).

Since OU noise constitutes the crucial part of the models studied within the current research study, we verify if indeed it produces the bimodal stationary state in the quartic potential that we are going to use in the proper model under investigation. Figure 1 presents time-dependent probability densities P(x,t) for a=1 and b=1 with ρ=0.05 and D= 40,000. Such an apparently large value of noise intensity was selected to assure the bimodality of the stationary state. Nevertheless, the large value of *D* is compensated by the small value of ρ, as in Equation (2), GWN is multiplied by the $\rho \sqrt{2D}$ factor. In Figure 1, the UCN approximation is depicted as the dot-dashed line. In the t→∞ limit, the time-dependent density approaches the bimodal shape predicted by the UCNA. For systems driven by OU noise, bimodality emerges due to competition between the deterministic restoring force and the correlated noise. For single-well potential, the deterministic force tries to push back all the probability mass to the origin, while the correlated noise is capable of producing a series of subsequent jumps in one direction. Such jumps allow particles to escape from the origin, resulting in the robust depletion in the probability mass and the emergence of bimodality. Consequently, for the quartic single-well potential, the bimodal stationary state can be recorded.

### 2.2. Proper Model: Random Potentials

For the main model of the current research study, we assume that the potential is no longer fixed (V(x)), but it stochastically changes over time (V(x,t)) due to the action of symmetric Markovian dichotomous noise [1,42,43]. Following the ideas of [44] and [45], we explore the possibility of the dynamical emergence of multimodal stationary states. We consider both 1D and 2D random potentials.

#### 2.2.1. One-Dimensional Random Potentials

Following the line of investigation initiated in [44] and extended in [45], we verify whether the addition of Markovian dichotomous noise can induce stationary states with more than two modal values. We consider Equation (1) with the 1D randomly switching potential

(10) $$V\left(x,t\right)=\frac{b}{4}{\left[x-\xi_{DN}\left(t\right)\right]}^{4}+\frac{a}{2}{\left[x-\xi_{DN}\left(t\right)\right]}^{2},$$

where $\xi_{DN}\left(t\right)$ is the symmetric Markovian dichotomous noise with the mean switching rate γ taking two values ±Δ with equal probabilities, i.e., $\xi_{DN}\left(t\right)\in \left[-\Delta ,\Delta \right]$, $\langle \xi_{DN}\left(t\right)\rangle =0$. The Markovian dichotomous noise has the exponentially decaying autocorrelation function

(11) $$\langle \xi_{DN}\left(t\right)\xi_{DN}\left(s\right)\rangle =\Delta^{2}\exp\left(-2\gamma \left|t-s\right|\right),$$

with the correlation time ${\left(2\gamma \right)}^{-1}$; see Ref. [1].

The model described by Equation (1) with the random potential given by Equation (10) is studied numerically. First of all, in order to verify that the stationary state has been reached, the interquartile widths and standard deviations are studied. For all considered sets of parameters for sufficiently large *t* (t>50), interquartile widths and standard deviations take their stationary values, and they are parallel to the axis of abscissas.

**Figure 2:**
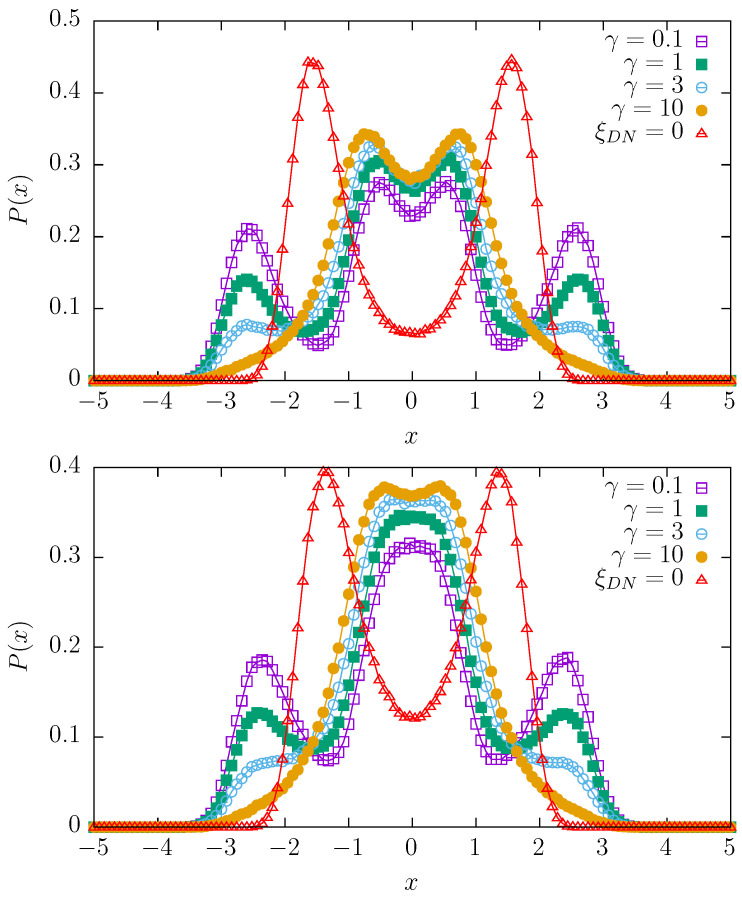
Stationary densities for various switching rates γ of the dichotomous noise with Δ=1 for a=0 and b=1 (**top** panel), and a=1 and b=1 (**bottom** panel) with ρ=0.05 and D= 40,000.

**Figure 3:**
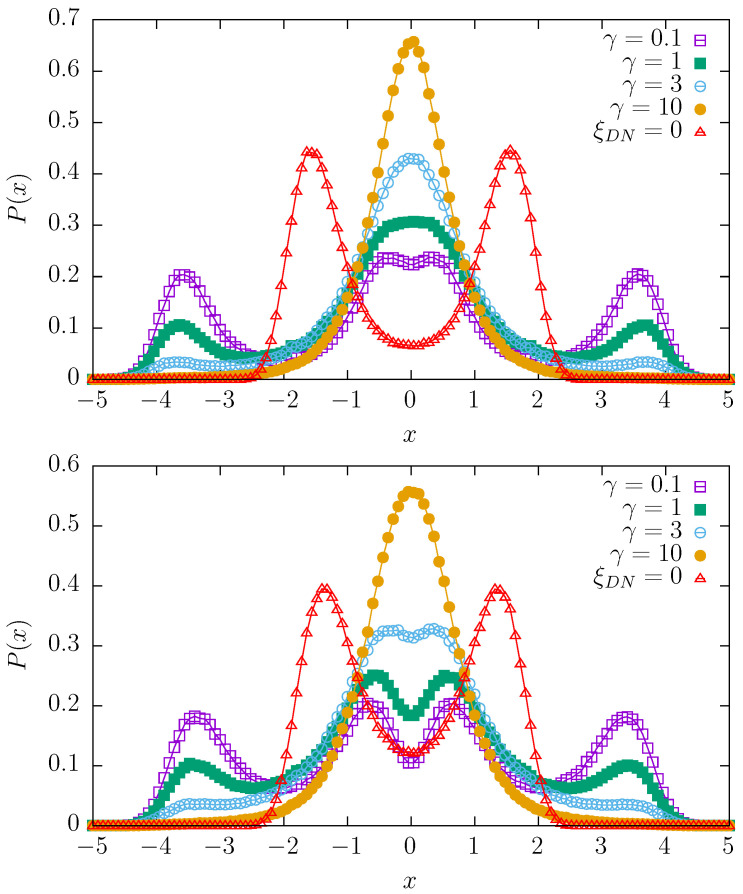
The same as in Figure 2 for Δ=2.

**Figure 4:**
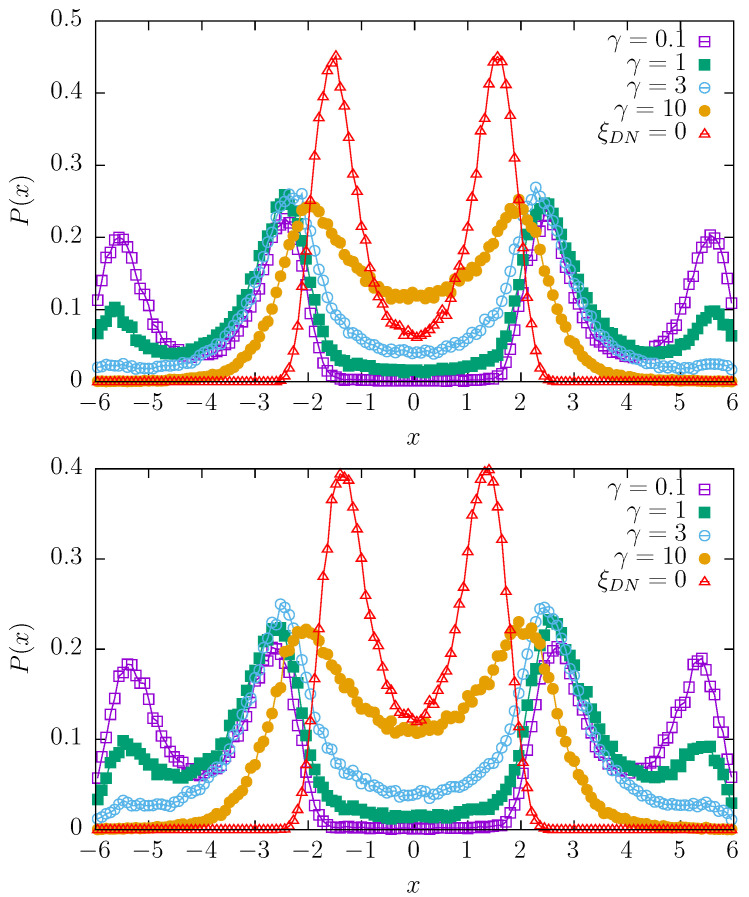
The same as in Figure 2 for Δ=4.

Figure 2, Figure 3 and Figure 4 present stationary states for various switching rates γ and various values of the dichotomous noise Δ∈{0,1,2,4}. The Ornstein–Uhlenbeck noise is characterized by ρ=0.05 with D= 40,000, which is the basic choice for the random potentials model; see Figure 1. In every figure, the top panels correspond to the (purely) quartic potential, i.e., $V\left(x,t\right)={\left(x-\xi_{DN}\right)}^{4}/4$ while bottom panels correspond to the mixture of quartic and parabolic potentials, i.e., $V\left(x,t\right)={\left(x-\xi_{DN}\right)}^{4}/4+{\left(x-\xi_{DN}\right)}^{2}/2$. In every plot, the solution corresponding to Δ=0 is plotted with red triangles and schematically labeled as $\xi_{DN}=0$. For the mixture of quartic and parabolic potentials (bottom panels of Figure 2, Figure 3 and Figure 4), this solution is the stationary solution of the model studied in the final part of Section 2.1; see Figure 1. Therefore, in order to show how the action of the dichotomous noise changes the stationary states, the stationary, reference state from Figure 1 is also included in the bottom panels of Figure 2, Figure 3 and Figure 4. It is worthy to mention that the results for γ<0.1 are practically indistinguishable from the results for γ=0.

From Figure 2, Figure 3 and Figure 4, one can conclude that for the appropriate choice of parameters, in comparison to the reference solution corresponding to the absence of dichotomous noise, the number of modes can be increased or decreased. In particular, one observes bimodal, trimodal, and four-modal stationary states. It is also possible to produce a unimodal stationary state. Interestingly, an increase in Δ, i.e., the values of the dichotomous noise, non-trivially changes the shapes of the stationary densities. The action of the dichotomous noise is not limited only to the shifting in the modes of the stationary densities outwards but also affects the modality of the stationary states. For instance, for Δ=2 with γ=10, the stationary states are unimodal (see Figure 3), while for Δ=1 and Δ=4, they are bimodal (see Figure 2 and Figure 4).

**Figure 5:**
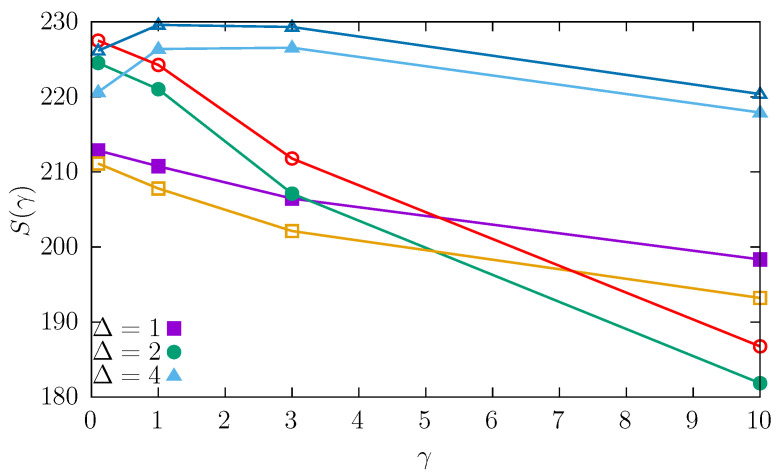
Shannon entropy S(γ) as a function of the switching rate γ, corresponding to the stationary states from Figure 2, Figure 3 and Figure 4. Full symbols correspond to a=0 and b=1 (pure quartic potential), while empty symbols correspond to a=0 and b=1 (quartic potential with harmonic addition); see Equation (10). Different curves show the results for different values of the dichotomous noise Δ. Lines are drawn to guide the eye only.

To characterize the ordering role of the dichotomous noise, the Shannon entropy S=−∫P(x)lnP(x)dx corresponding to the steady states shown in Figure 2, Figure 3 and Figure 4 is calculated. Figure 5 shows the Shannon entropy as a function of the switching rate γ of the dichotomous noise. The different curves correspond to different values of Δ; see Equation (11). The full symbols correspond to the pure quartic potential (a=0 and b=1), while the empty symbols correspond to the quartic potential with harmonic addition (a=1 and b=1); see Equation (10). From Figure 2, Figure 3 and Figure 4, it can be seen that with the increase in γ, the stationary states become more localized as the level of multimodality decreases. Figure 5 confirms that as switching rate of the dichotomous noise increases, the system becomes more ordered as the Shannon entropy typically decreases. An analogous effect will be visible in the 3D case (see below).

#### 2.2.2. Two-Dimensional Random Potentials

Finally, the model in Section 2.2.1 with the combined action of the symmetric Markovian dichotomous and Ornstein–Uhlenbeck noises is extended to the 2D setup. We use the 2D quartic potential

(12) $$V\left(\vec{r},t\right)=V\left(x,y,t\right)=\frac{1}{4}{\left[{\left(x-\xi_{DN}^{1}\left(t\right)\right)}^{2}+{\left(y-\xi_{DN}^{2}\left(t\right)\right)}^{2}\right]}^{2},$$

where $\xi_{DN}^{1}\left(t\right)$ and $\xi_{DN}^{2}\left(t\right)$ are two independent symmetric Markovian dichotomous noises taking the same values ±Δ. In Equation (12), two independent dichotomous noises are required; otherwise, the potential does not attain full generality, i.e., it cannot take all possible configurations.

The motion in potential (12) is perturbed by the two-dimensional Ornstein–Uhlenbeck process $\vec{\eta }\left(t\right)=\left(\eta_{x}\left(t\right),\eta_{y}\left(t\right)\right)$ satisfying the following equation:

(13) $$\dot{\vec{\eta }}\left(t\right)=-\rho \nabla \left[\frac{1}{2}{\vec{\eta }}^{2}\right]+\rho \sqrt{2D}\vec{\xi }\left(t\right),$$

where $\vec{\xi }\left(t\right)$ is 2D Gaussian white noise with the correlation

(14) $$\langle \xi_{i}\left(t\right)\xi_{j}\left(t+\tau \right)\rangle =\delta_{ij}\delta \left(\tau \right).$$

Equation (13) naturally generalizes Equation (2) to the 2D situation and describes the motion in a 2D parabolic potential; therefore, $\vec{\eta }\left(t\right)$ is built from two independent Ornstein–Uhlenbeck noises following Equation (2). Analogously, like in Equation (10), the mean switching rates characterizing the dichotomous noises are the same for both noises, and they are set to γ.

Overall, the 2D motion in the potential V(x,y,t) given by Equation (12) is described by the set of Langevin equations

(15) $$\left\{\begin{array}{ccc} \dot{x}\left(t\right) & = & -V_{x}^{\prime }\left(x,y,t\right)+\eta_{x}\left(t\right)\\ \dot{y}\left(t\right) & = & -V_{y}^{\prime }\left(x,y,t\right)+\eta_{y}\left(t\right) \end{array}\right.,$$

which can be rewritten in the vector form

(16) $$\dot{\vec{r}}\left(t\right)=-\nabla V\left(\vec{r},t\right)+\vec{\eta }\left(t\right).$$

**Figure 6:**
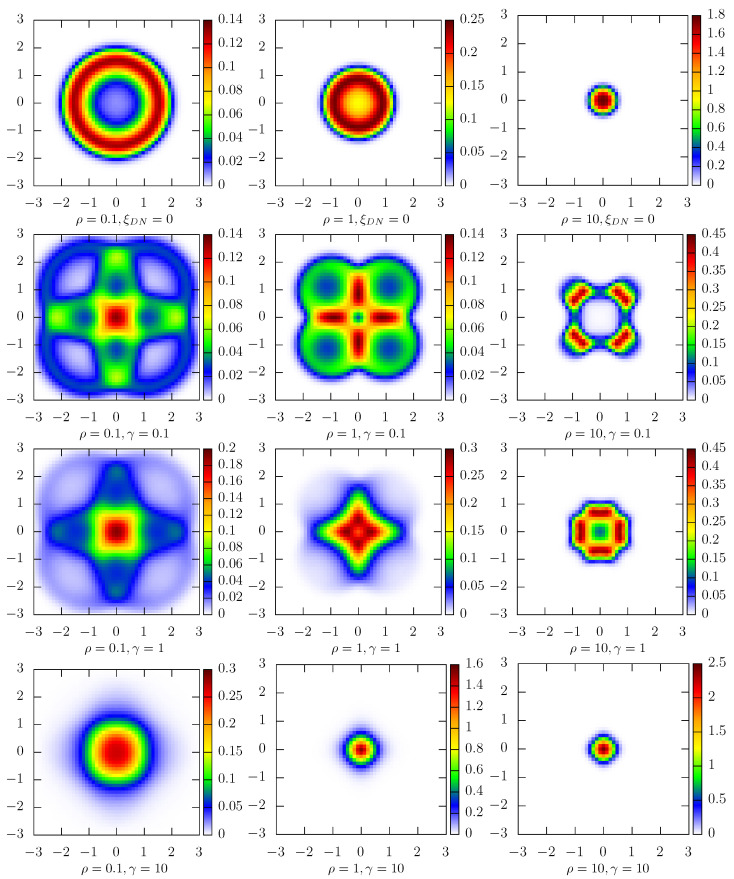
Stationary states for the 2D fixed and random potentials given by Equation (12). The top row depicts stationary states in the fixed single-well quartic potential ($\xi_{DN}\equiv 0$), while the remaining rows correspond to different switching rates of the dichotomous noise γ (γ∈{0.1,1,10}). Different columns correspond to different values of ρ (ρ∈{0.1,1,10}) in the Ornstein–Uhlenbeck noise. Other parameters: Δ=1 and D= 40,000.

**Figure 7:**
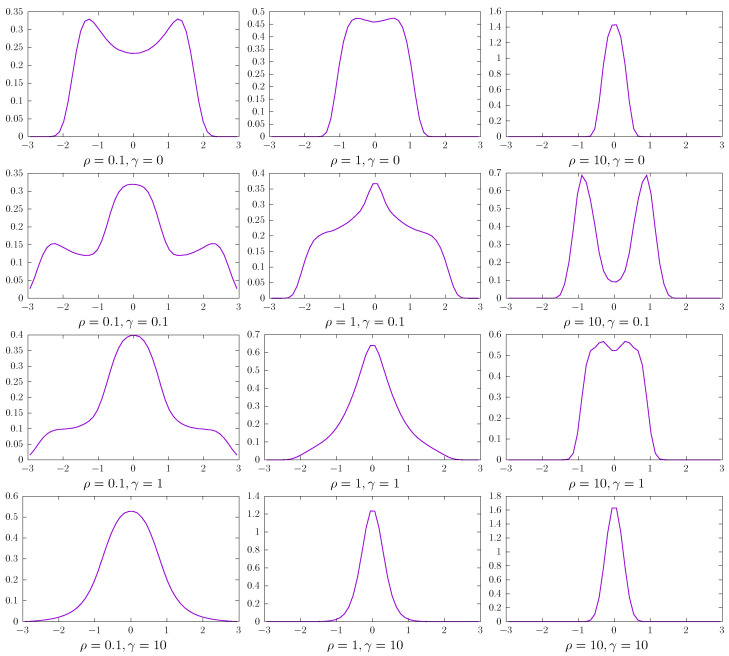
Marginal probability densities P(x)=∫P(x,y)dy corresponding to 2D stationary densities depicted in Figure 6.

Figure 6 and Figure 7 present results for the 2D potential given by Equation (12) with Δ=1. Figure 6 depicts 2D stationary densities P(x,y), while in Figure 7, marginal P(x)=∫P(x,y)dy densities are displayed. Due to the system symmetry, the P(y) densities have the same shape as P(x). The top row corresponds to the fixed single-well quartic potential $V\left(x,y\right)={\left(x^{2}+y^{2}\right)}^{2}/4$, i.e., the 2D random potential (12) without temporal modulation, which is used as the reference case. The subsequent rows correspond to the randomized 2D potential (12) with various values of the mean switching rate γ characterizing dichotomous noises. Finally, various columns show results for different ρ characterizing the additive Ornstein–Uhlenbeck noise. At the same time, the intensity *D* is kept constant, i.e., D= 40,000. Consequently, the top row generalizes the results of Ref. [13] to the 2D case. For $\xi_{DN}\left(t\right)=0$ the stationary states are spherically symmetric. For not too large ρ, i.e., long enough correlation time τ, the minimum of the stationary probability density function is at the origin. With the decrease in correlation time, the stationary density becomes unimodal. This transition is very well visible in the top row of Figure 7, which presents marginal probability density P(x). Additional action of symmetric Markovian dichotomous noises can induce dynamic multimodality with a non-trivial spatial pattern, as it is visible in the second and third rows of Figure 6. The minima of the 2D stationary states are recorded not only on the diagonals but also on the axes. Finally, for large switching rates γ (see the bottom row of Figure 6), the stationary states are unimodal and close to being approximately spherically symmetric. The shape of the marginal P(x) densities (see Figure 7) is derived from the complex shape of the 2D stationary states (see Figure 6). Analogously, as in the 1D case (see Figure 5), the Shannon entropy characterizing the stationary states decreases as the switching rate of the dichotomous noise increases.

## 3. Summary and Conclusions

The action of colored noise in fixed, single-well potentials can produce multimodal stationary states. Ornstein–Uhlenbeck noise is a Gaussian colored but Markovian noise often used to describe the interactions of dynamical systems with their environment. Its action for fine-tuned parameters of the model is capable of inducing the multimodality of the stationary states in single-well potentials. Bimodality emerges due to competition between the deterministic, restoring force and random, correlated fluctuations. Correlated noise is able to produce a persistent random force that can robustly shift particles from the minimum of the potential, transforming the maximum of the stationary density into a minimum. Consequently, it transforms a unimodal stationary density into a bimodal one. The transition between unimodal and bimodal stationary states can be studied numerically or by using approximate solutions for the stationary density. In this context, the unified colored noise approximation [12] seems to be especially useful.

The modality of stationary states can be further enhanced by the action of Markovian dichotomous noise, which can be used to randomize static potentials. For an overdamped motion in single-well potential wells driven by Gaussian white noise, it has been shown that the addition of Markovian dichotomous noise can induce dynamical multimodality [44]. The same effect can be observed for systems driven by Ornstein–Uhlenbeck noise. Since the stationary states for anharmonic stochastic oscillators driven by Ornstein–Uhlenbeck noise can be multimodal, the additional action of dichotomous noise can further increase the modality of the stationary states. Consequently, unimodal, bimodal, trimodal, and four-modal stationary states can arise. The randomization of the potentials can be easily extended to 2D setups, resulting in the emergence of additional modes of stationary states.

The problem of bimodal stationary states, along with unimodal–bimodal transitions, has been widely studied at the theoretical and abstract levels [14,15,16,17,18,40]. However, multimodal distributions occur in physics [13,46] and ecology/biology [47,48,49], among others. Therefore, the discussed model can potentially be used to justify the origin of bimodality in systems whose dynamics appear to be symmetric.

## Data Availability

The numerical, randomly generated data produced by the described model are available at https://doi.org/10.57903/UJ/6HEGHA (accessed on 26 February 2025). More information is available from the corresponding author upon reasonable request.

## Abbreviations

The following abbreviations are used in this manuscript:

DN	dichotomous noise
GWN	Gaussian white noise
OU	Ornstein–Uhlenbeck
UCNA	unified colored noise approximation
